# Preventing childhood scalds within the home: Overview of systematic reviews and a systematic review of primary studies

**DOI:** 10.1016/j.burns.2014.11.002

**Published:** 2015-08

**Authors:** Kun Zou, Persephone M. Wynn, Philip Miller, Paul Hindmarch, Gosia Majsak-Newman, Ben Young, Mike Hayes, Denise Kendrick

**Affiliations:** a Division of Primary Care, University of Nottingham, 13th Floor Tower Building, University Park, Nottingham NG7 2RD, UK; b Acute Medicine, Nottingham University Hospitals NHS Trust, City Hospital Campus, Hucknall Road, Nottingham NG5 1PB, UK; c Great North Children's Hospital, Research Unit Level 2, New Victoria Wing, Royal Victoria Infirmary, Queen Victoria Road, Newcastle upon Tyne NE1 4LP, UK; d NHS Clinical Research & Trials Unit, Norwich Medical School, University of East Anglia, Norwich NR4 7TJ, UK; e Child Accident Prevention Trust, Canterbury Court (1.09), 1-3 Brixton Road, London SW9 6DE, UK

**Keywords:** Scald, Prevention, Home, Children, Systematic review

## Abstract

**Objective:**

To synthesise and evaluate the evidence of the effectiveness of interventions to prevent scalds in children.

**Methods:**

An overview of systematic reviews (SR) and a SR of primary studies were performed evaluating interventions to prevent scalds in children. A comprehensive literature search was conducted covering various resources up to October 2012. Experimental and controlled observational studies reporting scald injuries, safety practices and safety equipment use were included.

**Results:**

Fourteen systematic reviews and 39 primary studies were included. There is little evidence that interventions are effective in reducing the incidence of scalds in children. More evidence was found that inventions are effective in promoting safe hot tap water temperature, especially when home safety education, home safety checks and discounted or free safety equipment including thermometers and thermostatic mixing valves were provided. No consistent evidence was found for the effectiveness of interventions on the safe handling of hot food or drinks nor improving kitchen safety practices.

**Conclusion:**

Education, home safety checks along with thermometers or thermostatic mixing valves should be promoted to reduce tap water scalds. Further research is needed to evaluate the effectiveness of interventions on scald injuries and to disentangle the effects of multifaceted interventions on scald injuries and safety practices.

## Highlights

• We performed an overview of published systematic reviews and a systematic review of primary studies evaluating the effectiveness of interventions to prevent scalds in childhood.
• There is little evidence that interventions are effective in reducing the incidence of scalds in children.
• There is no consistent evidence on the effectiveness of interventions on the safe handling of hot food or drinks nor improving kitchen safety practices.
• Education, home safety checks along with thermometers or thermostatic mixing valves is effective in reducing hot water temperature.

## 1 Introduction

Children are at particular risk of thermal injuries. Globally, thermal injuries are the 11th leading cause of death between the ages of 1 and 9 years and the fifth most common cause of non-fatal childhood injuries [1]. The majority of thermal injuries in the under-fives are scalds [2]. They are important as they can result in long term disability, have lasting psychological consequences and place a large burden on health care resources, with an estimated 19 million disability-adjusted life years lost each year [3]. The treatment of scalds is resource intensive. In the USA between 2003 and 2012, the average cost per hospital stay for scald injuries in the under-fives was between $40,000 and $50,000 [4]. The total cost of treating hot water tap scald injuries to children and adults in England and Wales in 2009 was estimated at £61 million [5].

Most scalds in the under-fives occur at home [2,6]. They are most commonly caused by hot liquids from cups or mugs, baths and kettles [8,9]. Bath water scalds are more likely to involve a greater body surface area especially in infants and toddlers and are more likely to undergo admission to hospital, transfer to specialist hospital or burns unit [8].

There are a number of systematic reviews that have synthesised the evidence on scald prevention interventions. However, most of them reviewed interventions to prevent a range of childhood injuries including scalds, some do not report conclusions specific to scald prevention and the remainder report conflicting conclusions [10–15]. One review [16] focussing on interventions specific to reducing thermal injuries in children concluded that there was a paucity of research studies to form an evidence base on the effectiveness of community-based thermal injury prevention programmes. A meta-analysis for which the searches were undertaken in 2009 found home safety education, including the provision of safety equipment, was effective in increasing the proportion of families with a safe hot tap water temperature, but there was a lack of evidence that home safety interventions reduced thermal injury rates or helped families keep hot drinks out of the reach of children [14].

There is therefore a need to consolidate evidence across existing reviews and update the evidence with more recently published studies to inform policy, practice, and the design and implementation of scald prevention. Overviews that synthesise all available evidence on a topic are more accessible to decision makers than multiple systematic reviews and can avoid uncertainty created by conflicting conclusions from different reviews, which may vary in scope and quality [17]. Overviews are useful where, as is the case for programmes to prevent scalds, there are multiple interventions for the same condition or problem reported in separate systematic reviews [18]. This paper presents the findings from an overview of reviews of childhood scald prevention interventions and a systematic review of primary studies to enable the most up-to-date information on scalds prevention interventions to be evaluated.

## 2 Methods

### 2.1 Literature search

We searched Cochrane Central Register of Controlled Trials (CENTRAL), Cochrane database of systematic reviews, MEDLINE, Embase, CINAHL, ASSIA, PsycINFO and Web of Science from inception to October 2012. We also hand-searched the journal Injury Prevention (March 1995–August 2012), abstracts of World Conferences on Injury Prevention and Control (1989–2012), reference lists of included reviews and primary studies, and a range of websites and trial registers for potentially relevant studies. No language limitation was applied.

### 2.2 Study selection

We included systematic reviews, meta-analyses, randomised controlled trials (RCT), non-randomised controlled trials (NRCT), controlled before-after studies (CBA) and controlled observational studies (cohort and case-control studies) targeting children aged 0–19 and their families to prevent unintentional scalds. The outcomes of interest were unintentional scalds, hot tap water temperature, use of thermometers to test water temperature, lowering boiler thermostat settings, use of devices to limit hot tap water temperature, keeping hot drinks and food out of reach, and kitchen and cooking practices. Potential eligible primary studies were identified from included systematic reviews by scanning references and further eligible primary studies were identified from additional literature searches of electronic databases and other sources. Titles and abstracts of studies were screened for inclusion by two reviewers. Where there was uncertainty about inclusion from the title or abstract the full text paper was obtained. Disagreements between reviewers were resolved by consensus-forming discussions and referral to a third reviewer if necessary.

### 2.3 Assessment of risk of bias and data extraction

We assessed the risk of bias in included systematic reviews and meta-analyses using the Overview Quality Assessment Questionnaire (QQAQ) [19]. The risk of bias of randomised controlled trials, non-randomised controlled trials and controlled before-after studies was assessed with respect to random sequence generation, allocation concealment, blinding of participants and personnel, blinding of outcome assessment, incomplete outcome data, selective reporting and other bias. The risk of bias in cohort and case-control studies was assessed using the Newcastle–Ottawa scale [20].

Data on study design, characteristics of participants (e.g. age, ethnicity, socio-economic group), intervention (content, setting, duration, intensity), and outcomes (injuries, possession or use of safety devices and safety practices) were extracted using separate standardised data extraction forms for reviews and primary studies.

Quality assessment and data extraction were conducted by two independent reviewers, with disagreements being resolved by consensus forming discussions and referring to a third reviewer if necessary.

### 2.4 Data synthesis

In view of the clinical heterogeneity between studies in terms of design, population, intervention and outcomes, data were synthesised narratively by types of outcomes including outcomes related to safe hot water temperature, safe handling of hot food and drinks such as keeping hot drinks and food out of reach of children, kitchen and cooking safety practices such as using cooker guards or keeping children out of kitchen and other outcomes related to scalds that could not be classified specifically.

## 3 Results

### 3.1 Study selection

**Fig. 1:**
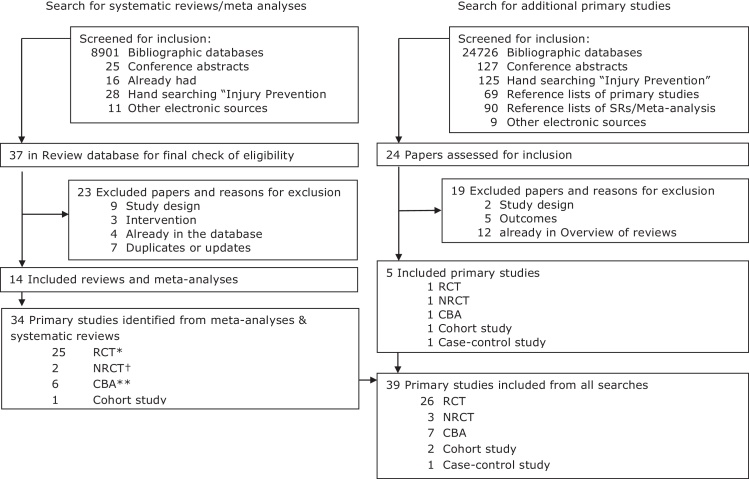
Selection of systematic reviews and primary studies for inclusion in the overview.

Fig. 1 shows the process of identification and selection of studies. Four meta-analyses (each of which also contained a narrative systematic review) and 10 systematic reviews and 39 primary studies were included in the overview. Of these primary studies, 34 were identified from published systematic reviews and meta-analyses and five were identified from the additional literature search (Table 1). Tables of excluded studies are available from the authors on request.

### 3.2 Study characteristics

Characteristics of included reviews are shown in Table 2. One review focused on community-based programmes to prevent scalds [16], while the remainder covered a range of injury mechanisms including but not specific to scalds. Only one review drew conclusions specific to scalds prevention interventions [16]. Two meta-analyses combined effect sizes from studies reporting safe hot tap water temperature [11,14] and one combined effect sizes from studies reporting keeping hot food and drinks out of reach [14]. Four systematic reviews narratively synthesised the evidence on the effect of interventions on scald injuries [12,13,15,16,21] and three on safe hot water temperature [10,12,15,21]. Seven systematic reviews reviewed the effectiveness of interventions on prevention of child injuries including burns and scalds, but did not make conclusions specific to scalds prevention [22–28].

The 39 eligible primary studies included 26 RCTs, 3 NRCTs, 7 CBAs, 2 cohort studies and 1 case-control study. The characteristics of included primary studies are show in Table 3. Most of the included studies employed multifaceted interventions including home safety inspections, education or counselling, provision of educational materials and safety devices. Included studies less commonly reported multifaceted home visiting programmes aimed at improving a range of child and maternal health outcomes, community multimedia campaigns, scald prevention education delivered through lectures or workshops, in clinical consultations, via specially designed computer programmes or other online educational material.

### 3.3 Risk of bias in reviews and in primary studies

Assessment of risk of bias is shown in Table 2 for reviews and Table 3 for primary studies. For reviews, OQAQ scores ranged from 1 to 7. For primary studies, 12 of the 26 RCTs (48%) had adequate allocation concealment, 10 (40%) had blinded outcome assessment and 14 (52%) followed up at least 80% of participants in each group. Of the nine NRCTs and CBAs, none had blinded outcome assessment, two (22%) followed up at least 80% of participants in each group and two (22%) had a balanced distribution of confounders between treatment groups.

### 3.4 Findings from included reviews and primary studies

Findings from included reviews are shown in Table 2 and from primary studies in Table 3.

### 3.5 Incidence of scalds

Six reviews reported interventions to prevent scalds from two primary studies [29,30]. No meta-analyses reported the effect of interventions on the incidence of scalds (Table 1). The first study [30], an RCT, reported significantly fewer self-reported scald injuries (validated against hospital and insurance records) two years after a school-based education programme in the intervention group (0.31%) than the control group (0.93%) (*p* < 0.05). The second study, a CBA, found a reduction in the number of scalds, particularly scalds from hot tap water and from hot cooking liquids being pulled from cooker tops, in the intervention areas over a 12 year period, but does not present similar data for the control area or the statistical significance of these findings [29].

### 3.6 Safe hot tap water temperature

Fourteen reviews reported the effect of interventions on safe hot tap water temperature from 26 primary studies and three primary studies reporting safe hot tap water temperature were identified from additional literature search (Table 1) [31–33]. Two meta-analyses combined effect sizes for having a safe hot tap water temperature, and both found a significant effect favouring the intervention group with pooled odds ratios of 2.32 (95% CI 1.46, 3.68) [11] and 1.41 (95% CI 1.07 to 1.86) [14] (Table 2). Three systematic reviews concluded there was a positive effect of interventions on safe hot water temperature from a narrative synthesis of the evidence [10,12,15].

Eighteen of the 29 studies clearly defined safe hot tap water temperature:

• less than or equal to 46 °C [34],
• less than 49 °C [33,35–41],
• less than or equal to 52 °C [31,42,43],
• less than or equal to 54 °C [32,44–47],
• less than or equal to 60 °C [48].

Eleven studies did not define safe hot tap water temperature (Table 3) [49–59].

Eleven studies reported significant effects favouring the intervention group for one or more outcomes related to safe hot tap water temperature including families having a safe hot water temperature, checking hot water temperature, and using engineering equipment to control hot water temperature (Table 3). This included nine RCTs [34,37,44,45,47,49,56,58,59], one CBA [43] and one cohort study [52]. Six studies reported significantly more families in the intervention than control group had a safe hot tap water temperature [34,37,43,44,47,49,59]. Five studies reported significantly more families in the intervention than control group checked or tested their hot tap water temperature [45,52,58], including one RCT specified using water temperature cards [49] and another using thermometers [56]. A cohort study found significantly more families exposed to the intervention lowered their hot water temperature than those not exposed to the intervention [52]. One RCT found significantly more families in the intervention than control group used spout covers for bath taps [56]. However, one CBA evaluating home safety checks, education and provision of bath water thermometers found significantly fewer families in the intervention group had a hot tap water temperature less than or equal to 52 °C than in the control group [42].

Most primary studies reporting significant effects on outcomes related to safe hot tap water temperature (including families having a safe hot tap water temperature, checking hot tap water temperature and using engineering equipment to control hot tap water temperature) employed multifaceted interventions. Three RCTs and one CBA provided safety education, a home safety assessment and safety equipment [37,43,44,49]. Two RCTs provided safety education and thermometers for checking water temperature [45,56]. One RCT provided education and thermostatic mixing valves fitted by qualified plumbers [34]. Two RCTs delivered educational lectures [47,59]. One RCT compared education plus supplying thermometers to supplying thermometers alone [58]. One cohort study compared families exposed to a multi-media scald prevention campaign with unexposed families [52].

Eighteen primary studies did not find a significant effect of interventions on outcomes related to safe hot tap water temperature including families having a safe hot water temperature, checking hot water temperature and using engineering equipment to control hot water temperature. These including 11 RCTs [31,35,36,38,40,42,48,50,53,55,57], two NRCTs [41,46], three CBAs [39,53,54], one cohort study [33] and one case-control study [32]. These studies evaluated integrated or individual interventions including home visits, home safety checks, counselling, safety education and offering safety devices.

### 3.7 Safe handling of hot drinks and food

Three systematic reviews and one meta-analysis looked into the effect of interventions on safe handling of hot drinks and food from seven primary studies [40,41,46,49,52,60,61]. Two more primary studies were identified through additional literature search [62,63] (Table 1). The meta-analysis estimated the pooled odds ratio for the effect of home safety education on keeping hot food and drinks out of reach; it failed to find a significant effect of the intervention (OR 0.95, 95% CI 0.61, 1.48) [14].

Of the nine studies, one RCT evaluated the effectiveness of education plus home safety assessments [60]. It found that significantly more families in the intervention group tested the temperature of food prepared in a microwave oven than the control families. The remaining eight studies (see Table 3) evaluating a range of interventions, including home safety education, tailored safety advice, home safety assessments, provision of discounted or free home safety equipment and exposure to Safe Kids Week champion, found no significant differences between the intervention and control groups. These included three RCTs [40,49,61], three NRCTs [41,46,62] and one CBA [63] and one cohort study [52].

### 3.8 Kitchen and cooking safety practices

Nine reviews reported the effectiveness of interventions on kitchen and cooking safety practices from 6 primary studies (Table 1) [41,52,56,60,64,65]. No meta-analyses reported pooled odds ratios related to kitchen and cooking practices. Two primary studies investigating interventions on kitchen and cooking safety practices were identified through additional literature search (Table 1) [32,62]. Two of the eight primary studies found significant effect of interventions. One RCT evaluating home safety education and home safety assessments reported that families in the intervention group were significantly more likely to have “childproofed” electrical heating devices in the kitchen (e.g. boiler, rice cooker) [60]. One NRCT evaluating home safety education, home safety assessments and burn and scald prevention workshops found that the intervention group were significantly more likely than the control group to have a “child-protected” cooker (not defined), and to have removed objects that a child could use to climb on to reach the sink [62].

However, the other six studies (Table 3) reporting on a variety of interventions including home safety education, home safety assessments, media campaigns, and free home safety equipment did not find any significant differences between the intervention and control groups in promoting kitchen and cooking safety practices. One RCT [65] evaluating the effectiveness of a school-based injury prevention programme found no significant differences between the practices of children in the intervention and control groups when cooking without an adult present. Another RCT [44] evaluating home safety education, home safety assessments and discount vouchers for safety equipment found no significant effect on keeping heating devices out of reach of children or for the use of stove guards. An RCT [56] assessing the effectiveness of an emergency department based home safety intervention found no significant effect on cooking on the back burners of cookers or turning pan handles towards the back of the cooker. An NRCT [41] evaluating providing tailored home safety education found no significant effect on keeping children away from the cooker or oven or on turning pan handles away from the edge of the cooker. One cohort study [52] evaluating Safe Kids Week 2001 found no significant differences between families who had been exposed to a media campaign on scald and burn prevention and controls for kitchen and cooking safety practices including cooking on the back burners of the cooker, keeping children out of the kitchen when cooking, turning pot handles to the back of the cooker and removing dangling cords of heating devices. A case-control study [32] investigating hazards in the homes of children who had presented with injuries from falls, burns, scalds, ingestions or choking found that no significant differences between cases and controls for having a cooker guard or not having dangling cords of heating devices.

### 3.9 Other scald-related outcomes

Eight reviews reported other scald-related outcomes such as burn safety scores which comprised a range of burn prevention behaviours such as pot handles left facing the edge of stove, not drinking tea/coffee or eating hot food when a child is on someone's lap, putting cool water in first when running a bath, or in some studies, undefined scald-related safety practices and undefined use of safety devices. No meta-analyses reported pooled odds ratios for any other scald-related outcomes. Four primary studies reported other scald-related outcomes. Two RCTs found significant effects on intervention groups from home safety education, home safety assessments and free home safety equipment on the burn safety scores (representing safer burn prevention practices) than the control groups [56,66]. One RCT found significantly more families in the intervention group made their homes safer after a television campaign, home safety advice, a home safety assessment check and advice on welfare benefits available to purchase safety equipment and local availability of equipment [64]. One CBA found no significant effect of a multi-faceted campaign (Hot Water Burns Like Fire) aimed at reducing the occurrence of scalds in children aged 0–4 years on scald prevention behaviours [51].

## 4 Discussion

This overview synthesised the largest number of primary studies evaluating child scald prevention interventions to date. Eligible studies were identified from comprehensive searches of published reviews, electronic databases, conference abstracts and other sources minimising the potential for publication and reporting bias. Rigorous procedures were used for study selection, quality assessment and data extraction. Our overview incorporated evidence from a spectrum of study designs including RCTs, NRCTs, CBAs, cohort studies and a case-control study to ensure maximum ascertainment of evidence in the field.

There was little evidence of the effect of scald prevention interventions on the incidence of scalds. We were able to find only two studies reporting scald occurrence, one of which reported a significant reduction in the incidence of scalds following a primary school-based injury prevention programme targeting school children and parents [30]. The second reported a reduction in the incidence of scalds following a community burn prevention programme comprising home safety education, home safety assessments, the promotion and installation of cooker guards and lowering tap water thermostat settings [29]. However, the statistical significance of the reduction in scalds was not reported.

There was more evidence that home safety interventions are effective in promoting safe hot tap water temperature with two meta-analyses and 11 primary studies reporting significant effects favouring the intervention group. Most studies with significant effects provided home safety education, home safety assessments and discounted or free safety equipment including thermometers and thermostatic mixing valves. We did not find any consistent evidence that home safety interventions were effective in promoting the safe handling of hot food or drinks, or kitchen and cooking safety practices, but the number of studies reporting these outcomes was small. In addition, there was wide variation and a lack of standardisation in the tools used to measure these outcomes, which hampered evidence synthesis in general and meta-analysis in particular.

There are several limitations of the review. First, there was considerable heterogeneity in the content of interventions of included studies and most studies used multifaceted interventions, hence it was not possible to attribute treatment effects to specific components of interventions. Care needs to be taken in interpreting the effects of interventions on hot tap water temperature due to the varying definitions of a “safe” temperature used by different studies and some studies not providing the definition they used. In addition, the temperature defined as “safe” has reduced over time, with more recent studies using a lower temperature than older studies. Consequently it is possible that the interventions in our review may not reduce hot tap water temperatures to levels that would now be considered sufficient to substantially reduce the risk of scalds. There was also considerable variation in study populations across included studies, making it difficult to ascertain if interventions would benefit specific groups of children or families to a greater degree. The vast majority of included studies were undertaken in high income countries, limiting the generalizability of our findings to low and middle income countries. The risk of bias varied across studies, but up to half of the RCTs had adequate allocation concealment, blinding of outcome assessment and follow up of at least 80% of participants in each group. For the NRCTs and CBAs, none had blinded outcome assessment, and only one in five had follow up of at least 80% of participants in each group or balance of confounding factors between groups.

The new evidence we found was consistent with the findings from the two published meta-analyses [11,14] and from the published narrative systematic reviews [10,12,15,21] which found home safety interventions were effective in promoting a safe hot tap water temperature. Our findings were also consistent with the previous meta-analysis and many systematic reviews that failed to find evidence that home safety interventions improved other scald prevention practices or reduced the incidence of scalds.

Our finding that most studies which were effective in promoting a safe hot tap water temperature included home safety education, home safety assessments and free or discounted safety equipment differed from that of the review by Pearson and colleagues [27]. This review focussed on home safety assessments, with or without the provision of safety equipment. Since publication of that review, two new studies have demonstrated significant effects favouring the intervention group [34,37], both of which provided free home safety equipment. In addition, our review included a wider range of interventions and these differences may partly account for the apparent inconsistency in our findings.

Although this review focussed on interventions that could be delivered in health and social care settings, other engineering or legislative approaches may be beneficial in reducing scalds. A recent trial evaluating thermostatic control of social housing estate boiler houses with daily sterilisation demonstrated significant reductions in hot tap water temperature [67]. Legislative changes such as those requiring new boiler thermostats to be set at lower temperatures or requiring thermostatic mixing valves in domestic settings are likely to be cost-effective. An economic analysis of one of the trials included in this overview found home safety education plus fitting of thermostatic mixing valves as part of bathroom refurbishment of social housing stock saved £1.41 ($2.35, €1.70) for every £1 ($1.65, €1.20) spent [68]. A recent Canadian study evaluating legislation to lower thermostat settings on domestic hot water heaters accompanied by yearly educational information provided to utility company customers estimated cost savings of C$531 per scald averted [69]. It is therefore important that scald prevention strategies encompass other engineering and legislative approaches as well as educational ones.

The paucity of evidence we found highlights the need for research to investigate the effect of interventions on reducing the incidence of childhood scalds in the home, the safe handling of food and drinks, and safe kitchen and cooking practices. Researchers should use existing validated tools to measure these outcomes wherever possible to facilitate evidence synthesis and meta-analysis. In terms of helping households to have a “safe” hot tap water temperature, further analyses are required to disentangle the effects of providing home safety education, thermometers, home safety assessments and thermostatic mixing valves. Network meta-analysis has previously been used to good effect in synthesising the evidence for smoke alarms [70] and is likely to be helpful in this situation. Providers of child health and social care should provide education to reduce tap water scalds, along with thermometers or thermostatic mixing valves. Public health policy-makers and practitioners should develop and implement scald prevention strategies that encompass legislative, engineering and educational approaches to reduce scalds risk.

## Conflict of interest statement

None.

## Figures and Tables

**Table 1:** Eligible primary studies in the included systematic reviews.

Year	Author	Design	Reviews														Outcomes				
			Bass 1993	U.S. PSTF 1996	DiGuiseppi 2000	Elkan 2000	Towner 2001	Waters 2001	Lyons 2003	Turner 2004	Kendrick 2007 a	Guyer 2009	Pearson 2009	Parbhoo 2010	Turner 2011	Kendrick 2012	Scald injuries	Safe hot water temperature	Safe hot drinks and food	Safe kitchen and cooking	Other outcomes
Primary studies from reviews																					
Babul	2007	RCT											•		•	•		S	NS		
Barone	1988	RCT			•						•					•		NS			
Chow	2006	RCT														•			S	S	
Colver	1982	RCT					•	•	•							•				NR	S^*^
Gaffney	1996	CBA														•					NS
Georgieff	2004	CBA									•					•		NS			
Gielen	2002	RCT									•					•		NS			
Hendrickson	2002	RCT														•			NS		
Katcher	1989	RCT	•		•				•		•					•		NS			
Kelly	1987	RCT			•						•					•		NS			
Kendrick	2007	RCT														•				NS	
Kendrick	2011	RCT														•		S			
Kendrick	1999	NRCT														•		NS	NS		
King	2001	RCT							•		•	•	•		•	•		S			
Macarthur	2003	Cohort								•								NS	NS	NS	
Minkovitz	2003a^†^	RCT									•	•						NS			
Minkovitz	2003b^†^	CBA									•							NS			
Mock	2003	CBA									•					•		NR			
Nansel	2002	RCT														•		NS	NS		
Nansel	2008	NRCT														•		NS	NS	NS	
Paul	1994	RCT											•			•		NS			
Phelan	2011	RCT														•		S			
Posner	2004	RCT									•	•	•		•	•		S		NS	S
Reich	2011	RCT									•							NS			
Sangvai	2007	RCT													•	•		NR			
Schwarz	1993	CBA					•	•	•				•	•		•		S^*^			
Shapiro	1987	RCT			•						•					•		NR			
Swart	2008	RCT														•					S
Sznajder	2003	RCT														•		NS			
Thomas	1984	RCT	•		•			•	•		•					•		S			
Waller	1993	RCT		•		•		•	•		•					•		NS			
Williams	1988	RCT			•											•		S			
Ytterstad	1998	CBA					•			•	•					•	S				
Zhao	2006	RCT														•	S				

Primary studies from additional literature search																					
Carlsson	2011	NRCT																	NS	S	
Christakis	2006	RCT																NR			
Gomez-Tromp	2011	CBA																	NS		
LeBlanc	2006	Case-control																NS		NS	
Margolis	2001	Cohort																NS			

Notes: US PSTF: U.S. Preventive Services Task Force; for outcomes, S = significant effect favouring I group. S^*^ = Significant effect favouring control group, NS = non-significant, NR = no *p* value reported (outcomes with no *p* value were considered as non-significant in text description), ^†^ Both were reported in Minkovitz 2003.

**Table 2:** Characteristics and conclusions of included systematic reviews.

Authors	Narrative review or meta-analysis	Included study designs*	Review quality (OQAQ)	Dates searched	Language restriction	Age	Interventions	Major relevant conclusions of review
Bass et al. [10]	Narrative review	RCTs, NRCTs	4	May 1964 to July 1991	English	Not reported	Injury prevention counselling in primary care settings	The review supports the inclusion of injury prevention counselling as part of routine health supervision. Primary care-based injury prevention counselling studies indicate beneficial outcomes including decreased hot tap water temperature
DiGuiseppi and Roberts [11]	Narrative review and meta-analysis	RCTs	6	Date of inception to August 1998	None	0–19 years	Individual-level interventions delivered in clinical settings, including primary care and acute care	Individual-level interventions delivered in a clinical setting are a promising way to promote improvements in certain safety practices, including safe hot tap water temperature. Smaller effects were observed in higher quality trials
Elkan et al. [22]	Narrative review and meta-analysis	RCTs, NRCTs, CBAs	5	Date of inception to 1997	Not reported	All ages	British home visiting by health visitors or personnel with responsibilities within the same remit	There was evidence to suggest that home visiting was associated with reductions in the frequency of unintentional injury and prevalence of home hazards. No conclusions specific to scalds prevention
Guyer et al. [23]	Narrative review	Experimental, quasi-experimental	4	1996 to 2007	English	0–5 years	Counselling, safety equipment and home visits delivered by general practitioners, community health workers and paediatricians	Currently available research justifies the implementation of health interventions in the prenatal to preschool period–especially to prevent injuries. No conclusions specific to scalds prevention
Kendrick et al. [24]	Narrative review and meta-analysis	RCTs, NRCTs, CBAs	7	Date of inception to May 2005	None	0–19 years	Individual and group-based parenting interventions	There is some, but not conclusive, evidence that parenting interventions can have a positive effect on both home safety and childhood injury rates. No conclusions specific to scalds prevention
Kendrick et al. [14]	Narrative review and meta-analysis	RCTs, NRCTs, CBAs	7	Date of inception to May 2009	None	0–19 years	Home safety education and provision of safety equipment delivered by health or social care professionals, school teachers, lay workers or voluntary or other organisations in health care settings, schools and homes	There was a lack of evidence that home safety interventions were effective in reducing rates of thermal (fire and scald) injuries. Home safety interventions were effective in increasing having a safe hot tap water temperature
Lyons et al. [25]	Narrative review	RCTs, NRCTs, CBAs, ITS	7	Date of inception to 2002	None	All ages	Reduction of physical hazards in the home by community health workers, trained researchers/volunteers, general practitioners and paediatricians	There is very little high-grade evidence that interventions to modify the home physical environment affect the likelihood of sustaining an injury in the home. No conclusions specific to scalds prevention
Parbhoo et al. [26]	Narrative review	All designs	3	Not reported	English	0–15 years	Any strategy to reduce paediatric burns	The greatest evidence of effectiveness came from multipronged programs of caregiver education, public policy, community monitoring and legislation, supported by repetition of the prevention message in different forms. No conclusions specific to scalds prevention
Pearson et al. [27]	Narrative review	RCTs, NRCTs, CBAs, BAs	5	1990 to 2009	English	0–15 years	Supply and/or installation of home safety equipment and/or home risk assessments delivered by general practitioners, doctors, nurses, research assistants, paediatricians, community health workers and health visitors in various settings	Most studies found no significant reduction in injury with any intervention. No robust evidence for increased use of home safety equipment. Evidence for the effectiveness of home risk assessments alone is weak. The addition of the supply of home safety equipment does not appear to make a substantive difference to their effectiveness. No conclusions specific to scalds prevention
Towner et al. [15]	Narrative review	RCTs, NRCTs, CBAs, BAs	2	1975 to 2000	Not reported	0–14 years	Home inspection, modification and education delivered by paediatricians, local health staff, school staff and community outreach workers in any setting	There is little evidence that educational approaches alone have achieved any reductions in burn and scald injuries. There is little evidence that campaigns involving the distribution of devices to control hot water temperatures are an effective means of reducing water temperatures
Turner et al. [16]	Narrative review	NRCTs, CBAs	7	Date of inception to May 2007	Not reported	0–14 years	Community- based interventions to reduce burns and scalds in children	There is a paucity of research studies in the literature from which practitioners can draw an evidence-base regarding the effectiveness of community-based injury prevention programmes to prevent burns and scalds in children
Turner et al. [28]	Narrative review	RCTs	5	Electronic databases: date of inception to December 2009. Hand searching: May 2009 to May 2010	None	All ages	Physical adaptations to the home environment, including to the building fabric or ‘fixtures and fittings’, installation of grab rails, stair gates, fire-guards, cupboard locks, hot-water tap adaptations and lighting adjustments	None of the studies focusing on children demonstrated a reduction in injuries that might have been due to environmental adaptation in the home. There is very little high-grade evidence that interventions to modify the home physical environment affect the likelihood of sustaining an injury in the home. No conclusions specific to scalds prevention
USPSTF [13]	Narrative review	RCTs, NRCTs, CBAs	2	Searched to May 1995**	English	Not reported	Counselling in clinical settings to prevent household and recreational injuries	Periodic counselling of the parents of children on measures to reduce the risk of unintentional household injuries from hot tap water is recommended
Waters et al. [12]	Narrative review	All designs	3	Not reported	English	0–4 years	Education and environment modification targeted to individuals and communities and applicable to the Australian situation	Changes in legislation are effective in achieving lower hot tap water temperatures and decreasing injuries from scalds. Resource-intensive, large-scale campaigns that encompass a combination of strategies (education, product modification and regulations concerning hot water temperatures) are associated with significant reductions in scald injuries among young children, particularly the more severe injuries

* RCT = randomised controlled trial, USPSTF = United States Preventive Services Task Force, NRCT = non-randomised controlled trial, CBA = controlled before and after study, BA = uncontrolled before and after study, ITS = interrupted time series design.

** Search start date not specified.

**Table 3:** Characteristics of primary studies included in the review.

First author	Design and risk of bias^a^	Participants	Content of intervention	Scald injuries/Preventive measures N (%), Effect size (95%CI)
Babul [49]	RCT A–Y B–N F–N	Parents of new born infants at a general hospital serving mainly urban or suburban communities *N* = 600	*I*_1_: home visit from community health nurse, home safety check to identify hazards and teach parents how to remove or modify the hazards; free safety kit (smoke alarm, safety gate 50% discount coupon, table corner cushions, cabinet locks, blind cord windups, water temperature card, doorstoppers, electrical outlet covers, poison control sticker); instructional brochure targeting falls, burns, poisoning and choking; risk assessment checklist. *I*_2_: free safety kit (see I1). C: usual care.	Hot water temperature Safe hot water temperature (not defined) *I*_1_ = 121 (70%) *I*_2_ = 113 (69%) *C* = 80 (54%) *I*_1_ vs C OR = 2.65 (1.57, 4.46) *I*_2_ vs C OR = 2.21 (1.32, 3.69) Using temperature card *I*_1_ = 135 (78%) *I*_2_ = 104 (63%) OR = 2.38 (1.42, 3.97) Hot drinks and food safety Keeping hot drinks or food out of reach of children *I* = 325 (97%) *C* = 147 (99%) OR = 0.44 (0.10, 2.04)
Barone [50]	RCT A–N B–N F–N	Couples or individuals participating in well-child parenting classes *N* = 79	I: slides, handouts on burn prevention, bath water thermometer, hot water gauge, and usual safety education C: usual safety education	Hot water temperature Safe hot water temperature (not defined) *I* = 16 (40%) *C* = 15 (39%) OR = 1.02 (0.41, 2.53)
Carlsson [62]	NRCT B–U F–N C–N Intervention group had higher rate of child injuries than control group at baseline	Mothers with low educational level with 4–7-month-old babies attending two child health care centres *N* = 99	I: 30–60 min workshop discussing burn and scald prevention and a 1 h home visit offering individual-based information focusing on problem described by mothers and solutions and suitable actions to take regarding child injury prevention in the home C: usual care	Hot drinks and food safety Electrical cords or iron or coffee and water heating appliances not within reach of children *I* = 37 (95%) *C* = 23 (74%) OR = 4.8 (0.5, 49.2) Kitchen and cooking safety Cooker child protected *I* = 25 (64%) *C* = 10 (32%) OR = 3.08 (1.1, 8.7) Cooker securely anchored *I* = 21 (54%) *C* = 9 (29%) OR = 2.3 (0.8, 6.6) Cooker door secured *I* = 24 (62%) *C* = 16 (52%) OR = 1.2 (0.4, 3.3) Climbing possibilities to sink removed *I* = 30 (77%) *C* = 12 (39%) OR = 4.4 95%CI 1.5, 13.1
Chow [60]	RCT A–Y B–U F–N	Families in two districts of Hong Kong with children under 3 years admitted to hospital with an unintentional injury *N* = 170	I: educational materials, 4 quarterly home visits with active guidance on injury prevention and regular monthly telephone follow-ups with no scheduled visits from trained home visitors C: educational materials on injury prevention, and 2 assessment only visits	Hot drinks and food safety Significantly more intervention group families tested temperature of micro-waved food. *p* = 0.05 Figures not reported Kitchen and cooking safetySignificantly more intervention group families using child-proofed boilers and rice cookers and electrical heating devices. *p* = 0.05. Figures not reported *p* Values come from Chan [71] and Cooper et al. [70]
Christakis [31]	RCT A–Y B–Y F–Y	Parents of children < 11 years attending clinics in the previous 3 years *N* = 887	*I*_1_: web-based safety information for parents plus health care provider notification of safety topics parents had expressed interest in on-line and information *I*_2_: health care provider notification I_3_: web-based safety information for parents C: usual	Hot water temperature Hot water temperature < 51.6 °C *I*_1_ = 23 (13%), *I*_2_ = 24 (13%), *I*_3_ = 25 (12%), *C* = 14 (7%). No *p* value reported
Colver [64]	RCT A–U B–U F–N	Families with children < 5 years attending child health clinics, day nurseries, nursery classes and a toddler group in deprived area (*n* = 80)	I: encouraged to watch TV safety campaign; home visit; advice on benefits to obtain safety equipment and local availability of safety equipment. C: encouraged to watch TV safety campaign	Kitchen and cooking safety In group I, 7 family had cooker guards obtained and fitted No *p* value reported Other scald outcomesMade home safer *I* = 22 (60%) *C* = 4 (9%)
Gaffney [51] Abstract only available	CBA B–U F–U C–U	Populations of unspecified control and intervention areas (N not reported)	I: multi-faceted community campaign to reduce risk factors and the rate of hot water scalds in children aged 0–4 years C: no campaign	Other scald outcomes No changes in use of scald limiting products and preventive behaviours (undefined). No figures or P values reported
Georgieff [39]	CBA B–U F–N C–N Intervention group had higher percentage of single parents than control group at baseline	Children < 3 years from 5 deprived wards N =92	*I*_1_: awareness raising campaign including leaflets, a logo, a radio advert campaign, a bus advertising campaign, burns and scalds road shows (advice): free bath water thermometers (engineering) and hot tap water temperature testing by researchers *I*_2_: advice only C: no intervention	Hot water temperature Mean temperature after intervention (°C) *I*_1_ = 26, *I*_2_ = 31, *C* = 35. Hot water outlet temperature > 49 °C *I*_1_ = 12 (46%), *I*_2_ = 19 (61%), *C* = 26 (74%) Hot water temperature ≤ 49 °C *I*_1_ = 3 (12%), *I*_2_ = 5 (16%), *C* = 5 (14%) Unsure if hot water outlet temperature is ≤ 49 °C *I*_1_ = 11 (42%), *I*_2_ = 7 (23%), *C* = 4 (11%) Checks water temperature with elbow or thermometer *I*_1_ = 19 (73%), *I*_2_ = 16 (52%), *C* = 15 (43%) Ever put child into bath without checking water temperature *I*_1_ = 0 (0%), *I*_2_ = 0 (0%), *C* = 2 (6%) Owns TMV's *I*_1_ = 6 (29%), *I*_2_ = 0 (0%), *C* = 0 (0%) Uses thermostatic adjustment to reduce water temperature *I*_1_ = 5 (23%), *I*_2_ = 2 (6%), *C* = 2 (6%) Has left a run bath unattended *I*_1_ = 9 (35%), *I*_2_ = 7 (23%), *C* = 16 (46%) Uses tap cover or sits child away from tap *I*_1_ = 1 (4%), *I*_2_ = 1 (3%), *C* = 4 (11%) Does not put child in bath while bath running *I*_1_ = 5 (19%), *I*_2_ = 3 (10%), *C* = 4 (11%) Adult runs the bath *I*_1_ = 25 (96%), *I*_2_ = 25 (81%), *C* = 31 (89%) Child bathes with supervision *I*_1_ = 17 (65%), *I*_2_ = 13 (41%), *C* = 18 (51%) No *p* values reported for any outcomes
Gielen [35]	RCT A–U B–U F–U	First and second year paediatric residents and their patient-parents, low income population of parents of children aged 0–6 months (*n* = 187).	I: safety counselling by professional health educator; discounted home safety equipment during visit to Children's Safety Centre; home visit involving hazard assessment (targeting falls, burns and poisonings) and safety recommendations. C: safety counselling by professional health educator; discounted home safety equipment during visit to Children's Safety Centre	Hot water temperature Hot water temperature ≤ 48.9 °C *I* = 27 (47%), *C* = 27 (47%), no significant difference between groups. No *p* value reported
Gomez-Tromp [63]	CBA B–U F–U C–U	Children aged 9 to 13 years in 35 schools *N* = 1260	I: scalds prevention program consisted of seven lessons, a DVD, a workbook for each pupil and a downloadable teacher's manual C: waiting list	Hot drinks and food safety Children carrying hot water No significant difference between groups. No figures or *p* value reported
Hendrickson [61]	RCT A–N B–N F–Y	Mothers with children aged 1–4 years, predominantly Mexican/Mexican American *N* = 82	I: safety counselling from researchers; identification of home hazards; provision of safety equipment (door knob covers, smoke detectors or new batteries if smoke alarm already in situ, fire extinguisher, cabinet latches and outlet covers). C: none of the above	Hot drinks and food safety Keeping hot drinks or food out of reach of children *I* = 37 (97%), *C* = 36 (90%) OR = 4.11 (0.44, 38.57)
Katcher [45]	RCT A–U B–U F–N	Consecutive paediatric clinic clients randomised to two groups *N* = 697	I: counselling by paediatrician plus tap water thermometer and tap water safety literature C: counselling and tap water safety literature	Hot water temperature Hot water temperature < 54.4 °C *I* = 76 (76%) *C* = 28 (90%) OR = 0.34 (0.09, 1.22) Tested hot water temperature *I* = 122 (46%) *C* = 55 (23%) OR = 2.89 (1.97, 4.26) Boiler thermostat lowering *I* = 29 (14%), *C* = 17 (9%) No significant difference between groups. *p* Value not reported
Kelly [42]	RCT A–U B–Y F–N	Parents of 6 month old children attending primary care centre for well child care (*n* = 129)	I: three-part individualised safety course at well child care visits. C: routine safety education	Hot water temperature Hot water temperature < 52 °C *I* = 41 (75%) *C* = 34 (63%) OR = 1.72 (0.76, 3.91)
Kendrick [46]	NRCT B–N F–N C–Y	Children 3–12 months registered at 36 GP practices (*n* = 2119)	I: health visitor safety advice at child health surveillance; low cost equipment (stair gates, fire guards, cupboard and drawer locks, smoke alarms); home safety checks; first aid training. C: usual care	Hot water temperature Hot tap water temperature < 54 °C *I* = 103 (29%) *C* = 88 (25%) OR = 1.26 (0.90, 1.76) Hot drinks and food safety keeping hot drinks or food out of reach of children *I* = 191 (60%) *C* = 201 (63%) OR = 0.89 (0.65, 1.22)
Kendrick [24] (Risk Watch)	RCT A–Y B–N F–Y	Children aged 7–10 years in state funded primary schools *N* = 459	I: teachers trained by Fire Service Personnel to deliver teaching on falls; poisoning; and ﬁre and burns. Fire Service personnel provided free teaching resources. C: usual care	Kitchen and cooking safety Child never cooks without adult present *I* = 117 (72%) *C* = 141 (77%) OR = 0.90 (0.45, 1.82)
Kendrick [34]	RCT A–Y B–Y F–Y	Households with children < 5 years in social housing in disadvantaged communities *N* = 124	I: thermostatic mixer valve ﬁtted by qualified plumber and educational leaflets prior to and at the time of fitting C: usual care	Hot water temperature Bath hot tap water ≤ 46 °C *I* = 13 (81%) *C* = 2 (13%) RR = 6.09 (1.64, 22.62) Runs bath using cold water first *I* = 5 (13%) *C* = 11 (28%) RR = 0.55 (0.22, 1.39) Checks bath water temperature for every bath *I* = 32 (84%) *C* = 40 (100%) RR = 0.84 (0.73, 0.97) Baths are only run by adult *I* = 38 (95%) *C* = 38 (95%) RR = 1.00 (0.90, 1.10) Child baths always supervised by adult *I* = 32 (82%) *C* = 34 (85%) OR = 0.97 (0.79, 1.17) Child usually gets in bath after water has been run *I* = 39 (97%) *C* = 39 (97%) RR = 1.00 (0.90, 1.10) Child has been left alone in the bath *I* = 13 (33%) *C* = 8 (21%) RR = 1.11 (0.51, 2.41) Child has been left alone in bathroom while bath is running *I* = 12 (31%) *C* = 9 (23%) RR = 1.28 (0.62, 2.68)
King [44]	RCT A–Y B–Y F–Y	Children <8 years attending A&E for injury or medical complaint *N* = 1172	I: home safety check; information on correcting any deficiencies; discount vouchers for safety equipment; demonstrations of use of safety devices; information on preventing specific injuries provided by researcher. C: home safety check and safety pamphlet	Hot water temperature Hot tap water temperature ≤ 54 °C *I* = 257 (53%) *C* = 218 (46%) OR = 1.31 (1.14, 1.50)
LeBlanc [32]	Case-control NOS score = 7	Children aged ≤ 7 years presenting to an emergency department with injuries from falls, burns or scalds, ingestions or choking matched to children who presented during the same period with acute non-injury-related conditions. *N* = 692	Exposures of interest: tap water temperature higher than 54 °C, kettle or appliances with dangling cords, no stove guard	Exposures of interest Hot water temperature Tap water temperature >54 °C Cases = 140 (41%), controls = 154 (46%) OR = 0.85 (0.62, 1.15) Kitchen and cooking safety No stove guard Cases = 340 (99%) controls = 339 (98%) OR = 1.20 (0.37, 3.93) Kettle or appliances with dangling cords Cases = 9 (4%), controls = 14 (6%) OR = 0.64 (0.28, 1.49)
Macarthur [52]	Cohort NOS score = 6	Parents or guardians of children under 9 years N = 504	Exposed group: campaign (media, retail, and community partners) emphasising lowering hot water tap temperature, child safety in the kitchen, keeping hot drinks away from child) checking smoke alarms regularly. Unexposed group: none of the above	Hot water temperature Tested water temperature Exposed = 27 (12%), unexposed = 14 (6%) RR = 1.95 (1.05, 3.61) Lowered water temperature Exposed = 13 (6%), unexposed = 4 (2%) RR = 3.28 (1.09, 9.90) Hot drinks and food safety Let food cool before serving to children Exposed = 186 (74%), unexposed = 195 (77%) RR = 0.96 (0.87, 1.06) Kitchen and cooking safety Keeps children out of kitchen when cooking Exposed = 135 (54%), unexposed = 135 (54%) RR = 1.01 (0.86, 1.19) Cooks on back burners at stove Exposed = 102 (41%), unexposed = 119 (47%) RR = 0.86 (0.71, 1.05) Turns pot handles to the back of the stove Exposed = 21 (84%), unexposed = 214 (85%) RR = 0.99 (0.92, 1.07) Ensured electrical cords are not dangling from counter Exposed = 203 (81%), unexposed = 220 (87%) RR = 0.93 (0.86, 1.01)
Margolis [33]	Cohort NOS score =7	Low-income pregnant mothers and their infants under 2 years old in Durham, North Carolina *N* = 317	Exposed group: 2 to 4 home safety checks per month through the infant's first year of life providing parental education on child health and development and injury prevention Unexposed group: usual care (women who had sought prenatal care during the 9 months before the program's initiation)	Hot water temperature Hot water temperature < 49 °C Exposed group = 22 (42%), unexposed group = 10 (26%) OR = 2.1 (0.83, 5.09)
Minkovitz*[53]	RCT A–N B–Y F–N CBA B–N F–Y C–N Control group had fewer older mothers, fewer white families, fewer years of education, more single parents, lower income and less likely to own home than intervention group at baseline	RCT Children ≤ 3 years old *N* = 2235 CBA Children ≤ 3 years old *N* = 3330	I: “Healthy Steps Programme”, which included child safety, for the first 3 years of life including extended well child office visits (average 11 in first 2.5 years of life), home visits (average <2 in first 2.5 years of life), telephone help-line, parent groups, written information. Programme delivered by paediatricians and Healthy Steps Specialists (nurses, nurse practitioners, social workers and early childhood educators). C: conventional paediatric care	RCT: Hot water temperature Lowered temperature on water heater *I* = 519 (64.4%), *C* = 441 (60.4%), *p* = 0.11 CBA: Hot water temperature Lowered temperature on water heater *I* = 645 (54.25%), *C* = 516 (56.3%), *p* = 0.82
Mock [54]	CBA B–N F–N C–N Intervention group had higher percentage of safe responses than control group at baseline	Parents in different socioeconomic strata (SES) in the city of Mexico *N* = 1124	I: the upper SES group received clinic-based lectures and demonstrations on motor car and pedestrian safety, burn prevention, home safety and recreational safety. *I*_2_: the middle SES group received the intervention the same as *I*_1_, however, some of them received clinic-based counselling. I_3_:The lower SES group received injury prevention counselling at half-hour household visits C: usual care	Hot water temperature Tested hot water temperature *I*_1_ = 0 (0%), *I*_2_ = 0 (0%), I_3_ = 1 (4%), C_1_ = 2 (7%), C_2_ = 0 (0%), C_3_ = 0 (0%); only within group pre-post comparison *p* values reported
Nansel [40]	RCT A–Y B–U F–Y	Parents of children aged 6–20 months attending well child check *N* = 213	I: tailored computer generated safety advice in well child clinic. C: generic computer generated safety advice in well child clinic	Hot water temperature Hot tap water temperature ≤ 49 °C *I* = 25 (29%), *C* = 27 (30%) OR = 0.96 (0.50, 1.83) Hot drinks and food safety Keeping hot drinks or food out of reach of children *I* = 78 (92%), *C* = 84 (94%) OR = 0.66 (0.20, 2.18)
Nansel [41]	NRCT Participants randomly allocated to *I*_1_ and C arms and remainder allocated to *I*_2_ B–N F–N C–N *I*_2_ group were older, more likely to be Caucasian and had lower educational level than control group at baseline	Parents of children aged ≤ 4 years attending well child visits at 3 paediatric clinics with mainly low to middle income patients *N* = 594	*I*_1_: tailored injury prevention education *I*_2_: tailored injury prevention education and feedback to health care provider. C: general education	Hot water temperature Safe hot tap water temperature (≤ 49 °C) *I* = 42 (20%) *C* = 26 (27%) OR = 0.71 (0.40, 1.24) Hot drinks and food safety Keeps hot drinks or food out of reach of children *I* = 125 (95%) *C* = 55 (89%) OR = 2.65 (0.85, 8.25) Kitchen and cooking safety Turns pan handles away from edge of stove *I*_1_ = 7 (100%), *I*_2_ = 11 (92%), *C* = 12 (86%) OR combining both I arms: 3.00 (0.14 to 186.62) Keeps child away from stove or oven *I*_1_ = 4 (57%), *I*_2_ = 10 (83%), *C* = 11 (85%) OR combining both I arms: 0.51 (0.04 to 3.98)
Paul 1994	RCT A–U B–U F–N	Families with children aged 10 months to 2 years born at local rural hospital *N* = 205	I: home safety check; tailored education booklet; local safety equipment retail outlets identified, mail order addresses provided or equipment ordered through research team and made available at local hospital. C: none of the above	Hot water temperature TMVs kitchen/bathroom/laundry: no significant difference between intervention and control groups. No figures or *p* value reported Hot water outlets with safety taps in kitchen/bathroom/laundry: no significant difference between intervention and control groups. No figures or *p* value reported
Phelan [37]	RCT A–Y B–N F–Y	Pregnant women, aged 18 years and over, < 19 weeks gestation, attending prenatal practices N =355	I: home safety check; provision and fitting of free safety equipment (stair gates, non-slip matting under rugs, window guards, repair of stair handrails, cupboard/drawer locks, door knob covers, storage bins, socket covers, smoke detectors, CO detectors, stove guards, stove locks); safety advice handout. C: safety advice handout	Hot water temperature Hot tap water temperature ≤ 49 °C *I* = 109 (75%) *C* = 94 (64%) OR = 1.69 (1.03, 2.79)
Posner [56]	RCT A–Y B–Y F–N	Caregivers of children <5 years attending ED for home injury *N* = 136	I: home safety counselling by trained lay personnel; home safety kit (cupboard and drawer locks, socket covers, bath tub spout covers, non-slip bath decals, bath water thermometer, poison control centre number stickers, free small parts tester); home safety literature. C: home safety literature	Hot water temperature Use of water thermometer *I* = 43 (88%) *C* = 13 (28%) OR = 18.74 (6.45, 54.47) Has spout covers for bath taps *I* = 39 (80%) *C* = 18 (38%) OR = 6.28 (2.53, 15.61) Hot drinks and food safety Keeps hot drinks or food out of reach of children *I* = 34 (73.9%), *C* = 38 (80.6%) OR = 0.67 (0.25, 1.79) Kitchen and cooking safety Cooks on back burners of cooker *I* = 25/49 (%)*C* = 16/47 (%) OR = 2.02 (0.89, 4.60) Turns pan handles towards back of cooker *I* = 29 (57%) *C* = 23 (49%) OR = 1.59 (0.71, 3.59) Other scalds outcomes Burns safety score, Mean (SD) *I* = 76.0 (14.9), *C* = 68.4 (17.4), *p* < 0.03
Reich [38]	RCT A–Y B–Y F–Y	Low-income primiparous women *N* = 198	*I*_1_: educational intervention book during 3rd trimester and additional books when baby was 2, 4, 6. 9, and 12 months old via a home visit *I*_2_: books with the same illustrations but with different non-educational text on the same schedule as *I*_1_. C: did not receive any books	Hot water temperature Hot water temperature < 49 °C I vs C_1_ OR = 1.07 (SE 0.31), *p* = non-significant I vs C_2_ OR = 1.44 (SE 0.44), *p* = non-signifiant
Sangvai [36]	RCT A–Y B–Y F–N	Caregivers of children aged 0 to 5 years from 3 paediatric clinics at a health maintenance visit *N* = 319	I: safety counselling from physician and researcher, free safety equipment (smoke detectors, gun locks, cabinet locks, and water temperature cards) and brief educational hand-out for parents C: usual care	Hot water temperature Hot water temperature < 49 °C *I* = 6 (67%) *C* = 6 (86%) OR = 0.33 (0.03, 4.19)
Schwarz [43]	CBA (C) Allocation at census tract level A–U B–N F–N C–Y	Population of 9 census tracts, predominantly low income, urban, African-American *I* = 902 *C* = 1060	I: home safety check and modification; education in homes and at block and community meetings; provision of ipecac, smoke alarms and batteries, bath water thermometers, night lights, emergency centre number sticker and fridge sticker with information on preventing injury C: none of the above	Safety water temperature Hot water temperature <52 °C *I* = 570 (63.2), *C* = 776 (73.2), OR = 0.57 (0.46, 0.71)
Shapiro [58]	RCT A–U B–U F–Y	Women admitted to the maternity ward of 3 hospitals *N* = 604	I: Pamphlet about tap water scalds and thermometer for testing, plus a 1 min educational message summarising pamphlet C: pamphlet and thermometer	Hot water temperature Tested hot water temperature *I* = 155 (51%) *C* = 88 (29%) OR = 2.56 (1.83, 3.59) Lowered hot water temperature. Figures and *p* value not reported
Swart [66]	RCT A–N B–Y F–Y	Households with children under 10 years in low income communities *N* = 410	I: four times home safety checks plus advice on prevention of burns poisoning and falls; free safety devices (child proof locks and paraffin container safety caps). C: none of the above	Other scalds outcomes Burn hazard safety practice score Mean (SD) *I* = 2.5 (0.12) *C* = 2.9 (0.12), *p* = 0.021, Mean difference (95%CI) = −0.41 (−0.76, −0.07)
Sznajder [57]	RCT A–Y B–N F–Y	Socio-economically disadvantaged families when children aged 6–9 months, with medical or psychological difficulties which place them at high risk *N* = 100	I: free home safety kit (cupboard and drawer locks, door handle covers, furniture corner protectors, socket covers, non-slip bath mat, smoke alarm, poison control centre number stickers); home safety counselling by health professionals; safety leaflets. C: home safety counselling by health professionals; safety leaflets	Hot water temperature Hot water system has adjustable thermostat *I* = 5 (11%), *C* = 5 (10%) OR = 1.07 (0.29, 3.97) Safe hot tap water temperature (not defined) *I* = 0 (0%), *C* = 3 (6%), *p* value not reported
Thomas [47]	RCT A–N B–U F–Y	Parents attending well-baby classes *N* = 58	I: standard information and literature plus a lecture on burn prevention provided by nurse practitioners, leaflet on protecting home against fire, adjusting hot water settings and cost of smoke alarms at local stores, plus $7 discount coupon for a smoke alarm. C: standard information and literature	Hot water temperature Safe hot water temperature <54.4 °C *I* = 22 (76%) *C* = 6 (23%) OR = 10.48 (3.01, 36.47)
Waller [48]	RCT A–U B–U F–Y	A random sample of Dunedin area children ≤ 3 years taken from birth records *N* = 121	I: free plumbing advice, home visit to measure tap water temperature, discuss dangers of hot water in the home and how to reduce tap water temperature provided by nurses C_1_: no home visit C_2_: no home visit and no baseline data collection	Hot water temperature Hot water temperature < 60 °C *I* = 21 (41%) *C* = 31 (32%) OR = 1.49 (0.74, 3.01)
Williams [59]	RCT A–U B–N F–U	Pregnant women attending prenatal classes *N* = 74	I: 1 h lecture, handouts on burn prevention, usual safety education. C: usual safety education	Hot water temperature Safe hot water temperature (not defined) *I* = 22 (56%) *C* = 11 (31%) OR = 2.88 (1.10, 7.55)
Ytterstad [29]	CBA B–U F–Y C–N Control city had higher injury rates and educational level than intervention city at baseline	Children ≤ 5 years in the city of Harstad (intervention) and Trondheim (control) *N* = 14573 person years	I: promotion of tap water thermostat setting to 55 °C and of increased parental vigilance in putative burn risk situations C: none of the above	Scald injuries *I* = 42 (0.25%), *C* = 700 (0.73%). No *p* value reported Thermal injury severity and mechanism—severity of stove and tap water scalds reduced in intervention area but ﬁgures only reported for control area. No P values reported
Zhao [30]	RCT A–N B–Y F–Y	Primary school children aged 7 to 13 *N* = 5872, year 2000 *N* = 5880, year 2001	I: school based Health education to children and their parents on injury prevention including scalds prevention; safety storage of pot of hot water C: school based health education of other common childhood diseases	Scald injuries Self-reported scalds/burns 1 year after intervention *I* = 28 (0.88%), *C* = 25 (0.93%); not significant (*p* value not given) Self-reported scalds/burns 2 years after intervention *I* = 10 (0.31%), *C* = 18 (0.68%), *p* < 0.05

Risk of bias: A = allocation concealment, B = blinding of outcome assessment, F = follow up on ≥80% of participants, C = confounder balanced between groups, Y = adequate, N = not adequate, U = unclear.

a Bias of case-control and cohort studies was assessed using Newcastle—Ottawa quality assessment scale (NOS).

* Minkovitz [53] reported 1 RCT and 1 CBA.

## References

[1] World Health Organization. Burns Fact Sheet. No. 3652012 http://www.who.int/mediacentre/factsheets/fs365/en/.

[2] Peden M., Oyegbite K., Ozanne-Smith J., Hyder A.A., Branche C., Fazlur Rahman A., Peden M. (2008). World report on child injury prevention.

[3] IHME (2010). Evaluation IfHMa. The global burden of disease: generating evidence, guiding policy.

[4] American Burn Association (2013). National burn repository 2013 report.

[5] Communities and Local Government, Communities and Local Government (2008). Impact assessment of amending Part G (Hygiene) of the building regulations and the revision to approved document G.

[6] Delgado J., Ramirez-Cardich M.E., Gilman R.H., Lavarello R., Dahodwala N., Bazan A. (2002). Risk factors for burns in children: crowding, poverty, and poor maternal education. Inj Prev.

[8] Sambrook (1999). Burns and scalds accidents in the home.

[9] Drago D.A. (2005). Kitchen scalds and thermal burns in children five years and younger. Pediatrics.

[10] Bass J.L., Christoffel K.K., Widome M., Boyle W., Scheidt P., Stanwick R. (1993). Childhood injury prevention counseling in primary care settings: a critical review of the literature. Pediatrics.

[11] DiGuiseppi C., Roberts I.G. (2000). Individual-level injury prevention strategies in the clinical setting. Future Child.

[12] Waters E., Shield J., Nolan T., Green J., Elkington J., Moller J. (2001). Evidence-based health promotion: no. 4 child injury prevention. Public health.

[13] United States Preventive Services Task Force. In: Wilkins Wa, editor. Guide to clinical preventive services. 2nd ed. Baltimore, MD: United States Preventive Services Task Force; 1996.

[14] Kendrick D., Young B., Mason-Jones A.J., Ilyas N., Achana F.A., Cooper N.J. (2012). Home safety education and provision of safety equipment for injury prevention. Cochrane Database Syst Rev.

[15] Towner E., Dowswell T., Jarvis S. (2001). Updating the evidence. A systematic review of what works in preventing childhood unintentional injuries: Part 2. Inj Prev.

[16] Turner C., Spinks A., McClure R., Nixon J. (2004). Community-based interventions for the prevention of burns and scalds in children. Cochrane Database Syst Rev.

[17] Smith V., Declan D., Begley C.M., Clarke M. (2011). Methodology in conducting a systematic review of systematic reviews of healthcare interventions. BMC Med Res Methodol.

[18] Higgins JPT, Green S (editors). Cochrane Handbook for Systematic Reviews of Interventions Version 5.1.0 [updated March 2011]. The Cochrane Col)laboration, 2011. Available from www.cochrane-handbook.org.

[19] Shea B., Boers M., Grimshaw J., Hamel C., Bouter L. (2006). Does updating improve the methodological and reporting quality of systematic reviews?. BMC Med Res Methodol.

[20] Wells G.A., Shea B., O’Connell D., Petersen J., Welch V., Losos M. (2008). The Newcastle-Ottawa Scale (NOS) for assessing the quality of nonrandomized studies in meta-analyses. http://www.ohri.ca/programs/clinical_epidemiology/oxford.htm.

[21] Towner E., Dowswell T., Jarvis S. (2001). Updating the evidence. A systematic review of what works in preventing childhood unintentional injuries: Part 1. Inj Prev.

[22] Elkan R., Kendrick D., Hewitt M., Robinson J.J.A., Tolley K., Blair M. (2000). The effectiveness of domiciliary health visiting: a systematic review of international studies and a selective review of the British literature. Health Technol Assess.

[23] Guyer B., Ma S., Grason H., Frick K.D., Perry D.F., Sharkey A. (2009). Early childhood health promotion and its life course health consequences. Acad Pediatr.

[24] Kendrick D., Barlow J., Hampshire A., Polnay L., Stewart-Brown S. (2013). Parenting interventions for the prevention of unintentional injuries in childhood. Cochrane Database Syst Rev..

[25] Lyons R.A., Sander L.V., Weightman A.L., Patterson J., Jones S.A., Lannon S. (2003). Modification of the home environment for the reduction of injuries. Cochrane Database Syst Rev..

[26] Parbhoo A., Louw Q.A., Grimmer-Somers K. (2010). Burn prevention programs for children in developing countries require urgent attention: a targeted literature review. Burns.

[27] Pearson M., Garside R., Moxham T. (2009). Preventing unintentional injuries among under-15s in the home - Report 1: Systematic reviews of effectiveness and cost-effectiveness of home safety equipment and risk assessment schemes. http://www.nice.org.uk/guidance/ph30/resources/preventing-unintentional-injuries-among-under-15s-in-the-home-review-of-effectiveness-and-cost-effectiveness2.

[28] Turner S., Arthur G., Lyons R.A., Weightman A.L., Mann M.K., Jones S.J. (2011). Modification of the home environment for the reduction of injuries. Cochrane Database Syst Rev.

[29] Ytterstad B., Smith G.S., Coggan C.A. (1998). Harstad injury prevention study: prevention of burns in young children by community based intervention. Inj Prev.

[30] Zhao C.H., Qiu H.S., Qiu H.X. (2006). Interventions to prevent accidental injuries in children between 7 and 13 years of age. Chin J Contemp Pediatr.

[31] Christakis D.A., Zimmerman F.J., Rivara F.P., Ebel B. (2006). Improving pediatric prevention via the internet: a randomized, controlled trial. Pediatrics.

[32] LeBlanc J.C., Pless I.B., King W.J., Bawden H., Bernard-Bonnin A.C., Klassen T. (2006). Home safety measures and the risk of unintentional injury among young children: a multicentre case-control study. Can Med Assoc J.

[33] Margolis P.A., Stevens R., Bordley W.C., Stuart J., Harlan C., Keyes-Elstein L. (2001). From concept to application: the impact of a community-wide intervention to improve the delivery of preventive services to children. Pediatrics.

[34] Kendrick D., Stewart J., Smith S., Coupland C., Hopkins N., Groom L. (2011). Randomised controlled trial of thermostatic mixer valves in reducing bath hot tap water temperature in families with young children in social housing. Arch Dis Child.

[35] Gielen A.C., McDonald E.M., Wilson M.E.H., Hwang W-T., Serwint J.R., Andrews J.S. (2002). Effects of improved access to safety counseling, products, and home visits on parents’ safety practices. Arch Pediatr Adolesc Med.

[36] Sangvai S., Cipriani L., Colborn D.K., Wald E.R. (2007). Studying injury prevention: practices, problems, and pitfalls in implementation. Clin Pediatr.

[37] Phelan K.J., Khoury J., Xu Y., Liddy S., Hornung R., Lanphear B.P. (2011). A randomized controlled trial of home injury hazard reduction: the HOME injury study. Arch Pediatr Adolesc Med.

[38] Reich S.M., Penner E.K., Duncan G.J. (2011). Using baby books to increase new mothers’ safety practices. Acad Pediatr.

[39] Georgieff K., Maw C. (2004). The Wakefield district burns and scalds prevention project. http://captcopy.net76.net/captcopy.net76.net/pdfs/wakefield.pdf.

[40] Nansel T.R., Weaver N., Donlin M., Jacobsen H., Kreuter M.W., Simons-Morton B. (2002). Baby, be safe: the effect of tailored communications for pediatric injury prevention provided in a primary care setting. Patient Educ Couns.

[41] Nansel T.R., Weaver N.L., Jacobsen H.A., Glasheen C., Kreuter M.W. (2008). Preventing unintentional pediatric injuries: a tailored intervention for parents and providers. Health Educ Res.

[42] Kelly B., Sein C., McCarthy P.L. (1987). Safety education in a pediatric primary care setting. Pediatrics.

[43] Schwarz D.F., Grisso J.A., Miles C., Holmes J.H., Sutton R.L. (1993). An injury prevention program in an urban African-American community. Am J Public Health.

[44] King W.J., Klassen T.P., LeBlanc J., Bernard-Bonnin A.C., Robitaille Y., Pham B. (2001). The effectiveness of a home visit to prevent childhood injury. Pediatrics.

[45] Katcher M.L., Landry G.L., Shapiro M.M. (1989). Liquid–crystal thermometer use in pediatric office counseling about tap water burn prevention. Pediatrics.

[46] Kendrick D., Marsh P., Fielding K., Miller P. (1999). Preventing injuries in children: cluster randomised controlled trial in primary care. Br J Med.

[47] Thomas K.A., Hassanein R.S., Christopherson E.R. (1984). Evaluation of group well-child care for improving burn prevention practices in the home. Pediatrics.

[48] Waller A.E., Clarke J.A., Langley J.D. (1993). An evaluation of a program to reduce home hot tap water temperatures. Aust J Publ Health.

[49] Babul S., Olsen L., Janssen P., McIntee P., Raina P. (2007). A randomized trial to assess the effectiveness of an infant home safety programme. Int J Inj Contr Saf Promot.

[50] Barone V.J. (1988). An analysis of well child parenting classes: the extent of parent compliance with health-care recommendations to decrease potential injury of their toddlers.

[51] Gaffney D. (1996). Preventing hot water scalds among young children in New South Wales. 3rd International Conference on Injury Prevention and Control.

[52] Macarthur C. (2003). Evaluation of safe kids week 2001: prevention of scald and burn injuries in young children. Inj Prev.

[53] Minkovitz C.S., Hughart N., Strobino D., Scharfstein D., Grason H., Hou W. (2003). A practice-based intervention to enhance quality of care in the first 3 years of life. J Am Med Assoc.

[54] Mock C., Arreola-Risa C., Trevino-Perez R., Almazan-Saavedra V., Zozaya-Paz J.E., Gonzalez-Solis R. (2003). Injury prevention counselling to improve safety practices by parents in Mexico. Bull World Health Org.

[55] Paul C.L., Sanson-Fisher R.W., Redman S. (1994). Preventing accidental injury to young children in the home using volunteers. Health Promot Int.

[56] Posner J.C., Hawkins L.A., Garcia-Espana F., Durbin D.R. (2004). A randomized, clinical trial of a home safety intervention based in an emergency department setting. Pediatrics.

[57] Sznajder M., Leduc S., Janvrin M.P., Bonnin M.H., Aegerter P., Baudier F. (2003). Home delivery of an injury prevention kit for children in four French cities: a controlled randomized trial. Inj Prev.

[58] Shapiro M.M., Katcher M.L. (1987). Injury-prevention education during postpartum hospitalisation (abstract). Am J Dis Child.

[59] Williams G.E. (1988). An analysis of prenatal education classes: an early start to injury prevention.

[60] Chow C.B., Chan C.C., Cheung W.L., Chan Y.C., Cheng J.C.Y., Luis P.K. (2006). Promoting a safer household environment: a volunteeer-based home visit program. Health care and promotion fund—HCPF.

[61] Hendrickson S. (2005). Reaching an underserved population with a randomly assigned home safety intervention. Inj Prev.

[62] Carlsson A., Bramhagen A.C., Jansson A., Dykes A.K. (2011). Precautions taken by mothers to prevent burn and scald injuries to young children at home: an intervention study. Scand J Publ Health.

[63] Gomez-Tromp M., Ben Meftah J., Gebhardt W., Prinsenberg T. (2011). Effect evaluation of a school based burn and scalds prevention programme in The Netherlands. Burns.

[64] Colver A.F., Hutchinson P.J., Judson E.C. (1982). Promoting children's home safety. BMJ.

[65] Kendrick D., Groom L., Stewart J., Watson M., Mulvaney C., Casterton R. (2007). Risk watch: cluster randomised controlled trial evaluating an injury prevention program. Inj Prev.

[66] Swart L., Van Niekerk A., Seedat M., Jordaan E. (2008). Paraprofessional home visitation program to prevent childhood unintentional injuries in low-income communities: a cluster randomized controlled trial. Inj Prev.

[67] Edwards P., Durand M.A., Hollister M., Green J., Lutchmun S., Kessel A. (2011). Scald risk in social housing can be reduced through thermostatic control system without increasing Legionella risk: a cluster randomised trial. Arch Dis Child.

[68] Phillips C.J., Humphreys I., Kendrick D., Stewart J., Hayes M., Nish L. (2011). Preventing bath water scalds: a cost-effectiveness analysis of introducing bath thermostatic mixer valves in social housing. Inj Prev.

[69] Han R.K., Ungar W.J., Macarthur C. (2007). Cost-effectiveness analysis of a proposed public health legislative/educational strategy to reduce tap water scald injuries in children. Inj Prev.

[70] Cooper N.J., Kendrick D., Achana F., Dhiman P., He Z., Wynn P. (2012). Network meta-analysis to evaluate the effectiveness of interventions to increase the uptake of smoke alarms. Epidemiol Rev..

[71] Chan C.C. (2004). Promoting a safer household environment: a volunteer based home visit program. 7th World Conference on Injury Prevention and Safety Promotion.

